# Immunotherapy – 2072. Therapeutic effect and higher safety profile for allergic asthma in Cuban patients with sublingual immunmotherapy using tropical domestic mite allergen vaccines

**DOI:** 10.1186/1939-4551-6-S1-P154

**Published:** 2013-04-23

**Authors:** Raúl Lázaro Castro Almarales, Mirta Alvarez Castello, Mercedes Ronquillo Díaz, Mayda González León, Alexis Labrada Rosado, José S Rodríguez Canosa, Bárbara Ivonne Navarro Viltres, Yunia Olivia Diaz, Maytee Mateo Morejón

**Affiliations:** 1 Allergens Department, National Center of Bioproducts (BIOCEN), Allergology and General Integral Medicine, La Habana, Cuba; 2 Allergology Department, University Hospital General Calixto García, Havana, Cuba; WAO Member; Cuban Society of Allergy, Asthma and Clinical Immunology, Member; Cuban Society of Immunology Member; 3 Allergen Service, University Hospital "General Calixto García", Cuba; 4 Docent Polyclinic "Pedro Fonseca", Havana, Cuba; Cuban Society of Integral General Medicine, Member; Cuban Society of Allergy, Asthma and Clinical Immunology, Member; Cuban Society of Immunology Member; 5 Cuban Society of Allergy, Asthma and Clinical Immunology, Mayabeque, Cuba; 6 Allergen Service, University Hospital General Calixto García, Havana, Cuba; WAO Member; Cuban Society of Allergy, Asthma and Clinical Immunology, Member; Cuban Society of Immunology Member; 7 Allergen Department, National Center of Bioproducts (BIOCEN), Mayabeque, Cuba; Cuban Society of Immunology, Member; Cuban Society of Allergy, Asthma and Clinical Immunology, Member; 8 Allergen, Biocen, Cuba

## Background

Subcutaneous allergen-specific immunotherapy is burdened with the risk of severe systemic reactions; therefore, sublingual administration route has been increasingly investigated worldwide. The goal was to assess the therapeutic effect and safety of allergen therapeutic vaccines of *Dermatophagoides pteronyssinus*, *Dermatophagoides siboney* and *Blomia tropicalis* House-Dust mites (VALERGEN, BIOCEN, Cuba) by sublingual route, in asthmatic patients.

## Methods

Three Double-Blind Placebo-Controlled clinical trials were performed in 40 patients each, showing asthmatic symptoms and positive predominant Skin Prick Test (SPT) to each mite, respectively. Half of subjects were randomized to placebo. Patients received the treatment consisting on sublingual drops with increasing daily doses for 3 weeks and maintenance doses (2000 BU) twice a week until 12 moths. Therapeutic effect was assessed after 6 and 12 months using symptoms/medication diary cards, peak expiratory flow (PEF) measures and skin sensitivity to investigated mites. Adverse reactions were classified using the World Allergy Organization scale.

## Results

The treatment reduced significantly (p<0.01) clinical symptoms (38%, CI95%: 33-44) and medication intake (26%, CI95%:21-32) with respect to placebo. The skin sensitivity to the allergens decreased also significantly (p<0.01). The allergen amount needed to induce a positive SPT increased 52-fold. PEF variability decreased also significantly (p<0.05). The treatment was considered effective in 77% of patients. A major advantage as compared to subcutaneous route was a remarked lower frequency of adverse effects. Local reactions were noted only in 0.43% of administrations. No systemic reactions were observed.

## Conclusions

Summarizing sublingual immunotherapy using VALERGEN vaccines is effective and safe in mite-sensitive asthmatic patients.

